# Ecthyma gangrenosum caused by *Staphylococcus aureus* in hematological malignancies: Case reports and literature review

**DOI:** 10.1097/MD.0000000000030070

**Published:** 2022-08-19

**Authors:** Yuka (Kudo) Nagata, Noritaka Sekiya, Kazuaki Fukushima, Masao Horiuchi, Noriko Doki

**Affiliations:** a Department of Infection Prevention and Control, Tokyo Metropolitan Cancer and Infectious Diseases Center, Komagome Hospital, Tokyo, Japan; b Department of Clinical Laboratory, Tokyo Metropolitan Cancer and Infectious Diseases Center, Komagome Hospital, Tokyo, Japan; c Department of Infectious Diseases, Tokyo Metropolitan Cancer and Infectious Diseases Center, Komagome Hospital, Tokyo, Japan; d Division of Hematology, Tokyo Metropolitan Cancer and Infectious Diseases Center, Komagome Hospital, Tokyo, Japan.

**Keywords:** ecthyma gangrenosum, hematological malignancies, *Staphylococcus aureus*

## Abstract

**Rationale:**

Ecthyma gangrenosum (EG) is a potentially life-threatening, systemic infection generally caused by *Pseudomonas aeruginosa*. Data on EG caused by *Staphylococcus aureus* in patients with hematological malignancies are scarce. The present case report aimed to describe the clinical features of EG caused by *S. aureus* in patients with hematological malignancies and to provide a comprehensive review of previous studies on the topic.

**Patient concerns:**

The first patient was a 61-year-old man with acute myeloid leukemia who presented fever and multiple lesions during chemotherapy. The second patient was a 47-year-old man with myelodysplastic syndrome who developed progressive erythematous necrotic plaques on his extremities and face.

**Diagnosis:**

Both cases were diagnosed as EG caused by *S. aureus*. While the first patient had concurrent methicillin-resistant *S. aureus* (MRSA) bacteremia, the second patient had positive results only for tissue culture of the skin lesion isolated methicillin-sensitive S. aureus.

**Interventions:**

Vancomycin was initiated with critical care to the first patient. Cefazolin was administered to the second patient for 3 weeks, followed by cephalexin for 1 week.

**Outcomes:**

The first patient died of a brain hemorrhage and multiple organ failure. The second patient was cured without relapse.

**Lessons:**

Of 18 patients in the previous and current studies with EG caused by *S. aureus*, 6 (33%) had an underlying hematological malignancy, and 10 (56%) had EG caused by MRSA. While 28% of the patients had positive blood cultures, all tissue cultures were positive. All 3 fatalities had concurrent bacteremia (MRSA caused two). EG caused by MRSA with concurrent bacteremia can be fatal, especially in patients with hematological malignancies. Although *S. aureus*-associated EG in patients with hematological malignancies is relatively uncommon, tissue cultures with an initial gram stain smear are essential for selecting appropriate empirical antimicrobials, including the coverage of *S. aureus*.

## 1. Introduction

Ecthyma gangrenosum (EG) is an uncommon, cutaneous condition most often seen in immunocompromised patients,^[1]^ among whom the mortality rate is 10% to 38%.^[2]^ Although *Pseudomonas aeruginosa* is the most common causative agent,^[1]^ various other organisms have been implicated.^[3]^ EG without preceding bacteremia may have a better prognosis than EG with concurrent bacteremia.^[4]^ Signs of a poor prognosis include prolonged neutropenia and delayed antimicrobial therapy.^[2]^ Few previous reports have described EG caused by *Staphylococcus aureus* since Turnbull and Parry^[5]^ reported the first case in 1981. Therefore, the clinical characteristics and prognostic data on this condition are limited, especially in patients with hematological malignancies who have a high risk of EG. The present case report aimed to describe the clinical characteristics of EG caused by *S. aureus* in patients with hematological malignancies. Also provided is a review of previous studies on the topic. The institutional review board of Tokyo Metropolitan Cancer and Infectious Diseases Center Komagome Hospital approved this study. Informed consent was obtained, and the head of the medical team and the institutional review board have responsibility for the anonymization of the patient.

## 2. Case reports

### 2.1. Case 1

**Figure 1.**
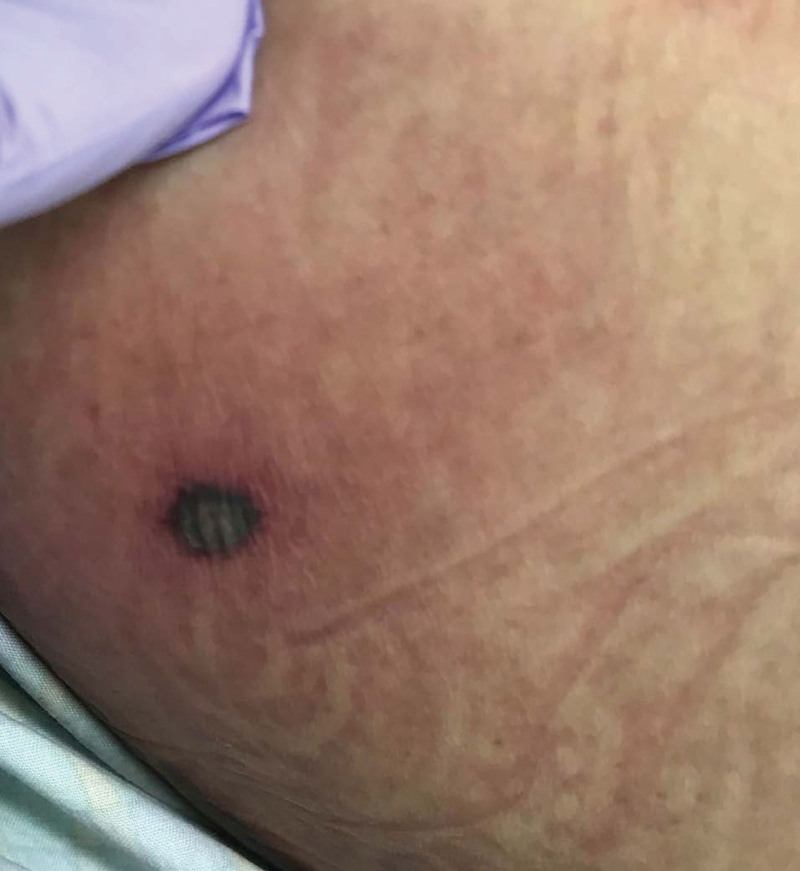
A skin manifestation of case 1. Erythema with central necrotic black eschar on the left flank.

A 61-year-old Japanese man with acute myeloid leukemia, well-controlled psoriasis vulgaris, and chronic atrial fibrillation was admitted for high-dose cytarabine as consolidation therapy after successful induction therapy. On hospital day 14, intravenous cefepime 2 g every 12 hours was initiated for febrile neutropenia. In a few days, an erythematous, edematous lesion with bullae developed on his chest under an electrocardiogram patch. Similar lesions developed over his face, flanks, and legs over the next 24 hours. Intravenous vancomycin 750 mg every 12 hours was added, and the central catheter was removed because a blood culture grew methicillin-resistant *S. aureus* (MRSA) and *Escherichia coli*. Infectious disease consultation was requested. The patient was intubated under sedation because his condition progressively deteriorated by septic shock and acute respiratory failure. His body temperature was 37.4°C, blood pressure 118/68 mmHg, pulse 140 beats per minute, and respiratory rate 18 breaths per minute on ventilatory support. Erythema with a central, black, necrotic eschar on his chest and similar facial lesions were noted. Some of these lesions were papules or vesicles (Fig. 1). The laboratory findings revealed that white blood cells were 20/L, hemoglobin was 6.9 g/dL, platelets were 13,000/L, and C-reactive protein was 39.9 mg/L. Transthoracic echocardiography revealed lower left ventricular systolic function and mild tricuspid regurgitation without vegetation. Pan-scan computed tomography revealed the absence of splenic infarct, renal infarct, psoas abscess, or other intraabdominal sites of infection. Initial gram stain smears showed gram-positive cocci in wound swab and catheter tip cultures, subsequently identified as MRSA. EG caused by MRSA with concurrent bacteremia was finally diagnosed. Although vancomycin with filgrastim resolved the bacteremia 4 days after the initiation of treatment, the former was switched to intravenous linezolid 600 mg every 12 hours because the skin lesions worsened and renal failure developed. The patient died from multiple organ failure and a brain hemorrhage despite neutrophil recovery 8 days after symptom onset.

### 2.2. Case 2

**Figure 2.**
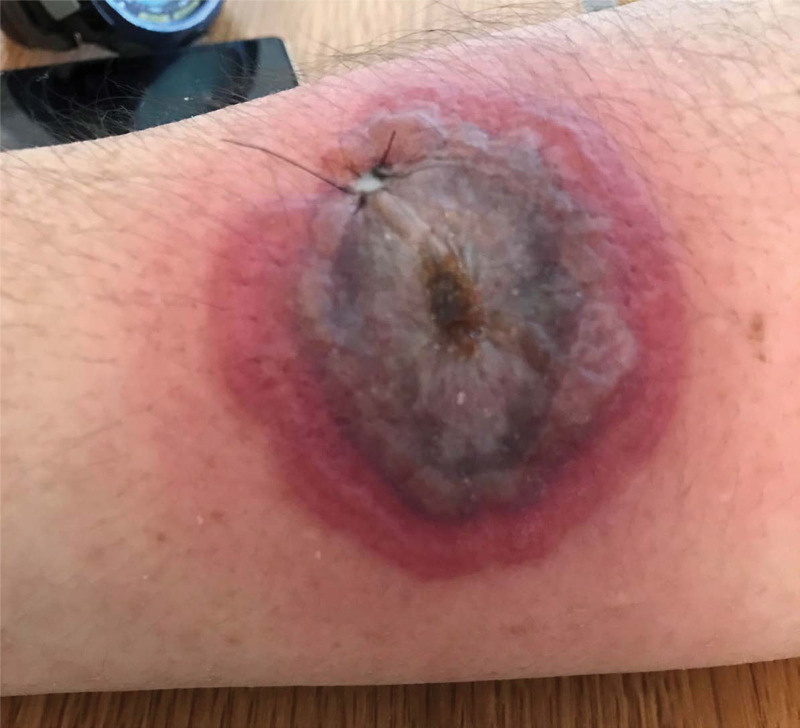
A skin manifestation of case 2. Formation of a hemorrhagic bulla on the right anterior forearm.

A 47-year-old Japanese man with myelodysplastic syndrome with multilineage dysplasia receiving blood transfusion support experienced a painless red macule on his left leg 3 weeks before admission. Two days before admission, progressive erythematous necrotic plaques developed on his extremities and face. Infectious disease consultation was requested. On examination, he was alert and oriented. His temperature was 40°C, blood pressure 121/58 mmHg, pulse 113 beats per minute, and respiratory rate 20 breaths per minute. A skin examination revealed multiple, deep, punched-out, painful ulcers with an erythematous halo on his left cheek and upper and lower extremities (Fig. 2). The laboratory findings included a white blood cell count of 5600/μL (neutrophils 64.5%, blasts 1%), hemoglobin 6.4 g/dL, platelets 12,300/μL, and C-reactive protein 16.4 mg/dL. Intravenous piperacillin-tazobactam 4.5 g every 6 hours and vancomycin 1.5 g every 12 hours were administered empirically. Transthoracic echocardiography revealed normal valvular function without vegetation, and pan-scan computed tomography was unremarkable. Blood cultures were negative. A punch biopsy specimen obtained from the edge of an ulcer on the left cheek showed no findings of the initial gram stain smears, finally growing methicillin-sensitive *S. aureus*. Histopathological examination revealed an ulcer and edema accompanied by dense infiltration of neutrophils into the dermis. Although vessel invasion, vasculitis, and necrosis were not present, extravasation of red blood cells was observed. These findings were compatible with EG. The antimicrobials were switched to intravenous cefazolin 2 g every 8 hours, and his erythematous halo gradually resolved. His punched-put ulcers became shallower with granulation tissue appearing over the 2-week period of hospitalization. After 21 days of intravenous antimicrobial therapy, he was discharged with additional sustained-release oral cephalexin 1000 mg twice daily for 7 days. No signs of recurrence were observed 1 year after finishing the treatment.

## 3. Discussion

We reported 2 cases of EG caused by *S. aureus* in patients with hematological malignancies. A comprehensive review of the English-language studies on EG caused by *S. aureus* published before 2021 was conducted using PubMed (https://pubmed.ncbi.nlm.nih.gov) and Google Scholar. The search terms were “*Staphylococcus aureus*” and “ecthyma gangrenosum.” Table 1 lists the results, including the present report. Six of 18 patients (33%) with EG caused by *S. aureus* had an underlying hematological malignancy, and 10 (56%) had EG caused by MRSA. While 72% of the patients had a negative blood culture (n = 13/18), all the tissue cultures (except those not analyzed) were positive. Three patients with concurrent bacteremia died (2 of the deaths were caused by MRSA). Our review indicated the following: *S. aureus*-associated EG in patients with hematological malignancies is uncommon; MRSA accounts for more than half of EG cases caused by *S. aureus*; and EG with MRSA bacteremia can have a poor prognosis.

**Table 1:** Ecthyma gangrenosum caused by *Staphylococcus aureus*.

Author	Age (yr)	Sex	Underlying diseases	Pathogen	Blood culture	Tissue culture	Neutropenia*	Treatment	In-hospital death
Hematological malignancies									
Turnbull and Parry^[5]^ (1981)	68	M	Erythroleukemia	MSSA	+	+	+	Oxacillin sodium: 4 wk	−
Turnbull and Parry^[5]^ (1981)	59	M	Waldenström’s macroglobulinemia	MSSA	−	+	NA	Oxacillin: duration unclear	−
Chang et al^[6]^ (2012)	35	F	ALL	MRSA	−	+	+	Vancomycin: duration unclear	−
Dassan et al^[7]^ (2015)	41	M	ALL	MRSA	+	+	+	Vancomycin: 2 wk	−
Our case 1	61	M	AML	MRSA	+	+	+	Died during treatment: antimicrobials for 8 d	+
Our case 2	47	M	MDS	MSSA	−	+	−	Antimicrobial therapy: 28 d	−
Others									
Nakai et al^[8]^ (2008)	40	M	Kidney transplant recipient	MRSA, *Pseudomonas aeruginosa*	−	+	+	Gentamicin: 19 d	−
Sen et al^[9]^ (2009)	69	M	COPD	MRSA	+	NA	−	Ampicillin/sulbactam, meropenem, teicoplanin: 19 d	+
Pechter et al^[10]^ (2012)	8 mo	F	No comorbidities	MRSA	−	+	−	Vancomycin: 21 d	−
Apstolova^[11]^ (2012)	59	M	FVL mutation	MRSA	−	+	−	Antimicrobials and surgical debridement: type of regimen and duration unclear	−
Ungprasert et al^[12]^ (2013)	40	M	HIV	MRSA	−	+	−	Vancomycin: 10 d; linezolid PO: 1 mo	−
Song et al^[13]^ (2015)	15 mo	F	No comorbidities	MSSA	−	+	−	Ceftriaxone → cefepime and clindamycin: 14 d	−
Ivanaviciene^[14]^ (2016)	54	F	Gastric adenocarcinoma	MSSA	−	+	+	Acyclovir, meropenem, vancomycin → intravenous oxacillin: 2 wk	−
Santhaseelan and Muralidhar^[15]^ (2017)	47	M	Chronic alcoholic	MRSA	−	+	−	Ceftriaxone → imipenem and amikacin with surgical debridement: duration unclear	−
Ulpiano Trillig et al^[16]^ (2019)	27	M	No comorbidities	*S. aureus*, GAS	−	+	−	Antimicrobial therapy: 7 d	−
Ulpiano Trillig et al^[16]^ (2019)	31	M	HIV	*S. aureus*, GAS	−	+	−	Antimicrobial therapy with surgical debridement: 14 d	−
Barry et al^[17]^ (2021)	19	M	CKD	MRSA	−	+	−	Linezolid without surgical debridement: 4 wk	−
Shah et al^[18]^ (2021)	62	M	Hypertension and hyperlipidemia	MSSA	+	+	−	Nafcillin: duration unclear	+

ALL = acute lymphoblastic leukemia, AML = acute myeloid leukemia, CKD = chronic kidney disease, F = female, FVL = factor V Leiden, GAS = group A *Streptococcus*, HIV = human immunodeficiency virus, M = male, MDS = myelodysplastic syndromes, MRSA = methicillin-resistant *S. aureus*, MSSA = methicillin-susceptible *S. aureus*, NA = not assessed.

* Neutropenia is defined as an absolute neutrophil count <1000 μL (equivalent to <1.0 × 10^^^9/L).

We note that EG due to *S. aureus* has different characteristics from disseminated systemic *S. aureus* infection, while these differences were not discussed in previous articles. Firstly, in clinical presentation, skin lesions of EG are rapidly progressive within 24 hours and lead to dermal necrosis and usually affect the groin, axilla, or trunk.^[19]^ On the other hand, most systemic *S. aureus* infections can be found preceding bacteremia, and skin lesions are located on the sole of a foot and palm of a hand, especially in patients with infective endocarditis.^[20]^ Secondly, each histopathological feature is distinct. While systemic *S. aureus* infections reflect septic emboli or micro-abscess without vasculitis, cutaneous vasculitis was generally seen in EG.^[20,21]^

The causative organisms in EG are detected by tissue and blood cultures. Although there is a publication bias, all tissue cultures performed in cases of EG caused by *S. aureus* returned positive for the pathogen. In contrast, only 5 cases of blood cultures returned positive. In the previous reports, 44% (74/167) of EG (any pathogen) was associated with positive blood culture results, while only 9% (4/44) of nonpseudomonal EG was associated with positive blood culture results.^[22]^ Therefore, tissue cultures can be crucial for diagnosing EG and selecting an appropriate empirical antimicrobial therapy, especially if a pathogen other than *P. aeruginosa* is suspected. In addition, initial gram stain smears of tissue culture can help determine an appropriate empirical antimicrobial agent, especially for cases with a negative blood culture. A past report mentioned that gram stain smears of exudate or material scraped from the base of a lesion can be valuable when choosing an antimicrobial therapy.^[23]^

EG with concurrent MRSA bacteremia is associated with poor outcomes. Although EG caused by *S. aureus* is uncommon in hematological malignancies, MRSA accounts for more than half of EG cases caused by *S. aureus*. Moreover, in our review, all 3 fatalities had concurrent bacteremia. Signs of a poor prognosis of EG include concurrent bacteremia, multiple lesions, delayed antimicrobial therapy, a poor response to appropriate antimicrobials, and prolonged neutropenia.^[2]^ The septicemic form of EG is associated with a poor prognosis and a high mortality rate of around 60%, in contrast to 15% for localized EG.^[24]^ Our review suggested that concurrent bacteremia can lead to a poor prognosis in EG caused by *S. aureus* and that *S. aureus* coverage should be considered when choosing an antimicrobial regimen for EG with hematological malignancies.

## 4. Conclusions

Although *S. aureus*-associated EG is uncommon in patients with hematological malignancies, awareness of causative agents other than *P. aeruginosa* is essential. A tissue culture with an initial gram stain smear is essential for selecting appropriate empirical antimicrobials. Additionally, EG caused by MRSA with concurrent bacteremia can be critical, especially for patients with hematological malignancies.

## Acknowledgments

We are indebted to Mr James R. Valera for his assistance with editing the article. We thank Dr Tsunekazu Hishima and all the staff at the Tokyo Metropolitan Cancer and Infectious Diseases Center, Komagome Hospital, for their excellent patient care.

## Author contributions

Conceptualization: Yuka (Kudo) Nagata, Noritaka Sekiya. Data curation: Yuka (Kudo) Nagata. Formal analysis: Yuka (Kudo) Nagata. Funding acquisition: None. Investigation: Yuka (Kudo) Nagata, Noritaka Sekiya, Kazuaki Fukushima, Masao Horiuchi. Methodology: Yuka (Kudo) Nagata, Noritaka Sekiya. Project administration: Yuka (Kudo) Nagata, Noritaka Sekiya. Supervision: Noritaka Sekiya, Noriko Doki. Validation: Yuka (Kudo) Nagata, Noritaka Sekiya. Visualization: Yuka (Kudo) Nagata. Writing—original draft: Yuka (Kudo) Nagata. Writing—review & editing: Yuka (Kudo) Nagata, Noritaka Sekiya, Kazuaki Fukushima, Masao Horiuchi, Noriko Doki. All authors meet the ICMJE authorship criteria.
